# Breast density and risk factors for interval and screen-detected breast cancers: a case–case analysis of the Breast Cancer Now Generations Study, UK

**DOI:** 10.1186/s13058-026-02355-z

**Published:** 2026-08-13

**Authors:** Louise Johns, Martina Brayley, Reuben Frost, Penny Coulson, Micheal Jones, Amy Berrington de Gonzalez, Montserrat Garcia-Closas

**Affiliations:** 1 Division of Genetics & Epidemiology, The Institute of Cancer Research, Brookes Lawley Building, 28 Oakleaf Avenue, London, SM2 5GP UK; 2 Cancer Epidemiology & Prevention Research Unit, The Institute of Cancer Research & Imperial College London, London, UK

**Keywords:** Breast cancer, Interval cancers, Risk factors, Mammographic density

## Abstract

**Background:**

Interval breast cancers (IBC), diagnosed between routine screening rounds, tend to have a worse prognosis than screen-detected breast cancers (SDBC). Identifying risk factors for IBCs is critical for improving early detection and developing risk-stratified screening strategies to reduce their incidence. We evaluated associations of breast density, reproductive, hormonal, lifestyle and medical factors with IBC compared to SDBC in a large UK cohort.

**Methods:**

Analyses included 1,940 women diagnosed with breast cancer between 2004 and 2018 after enrolment in the Breast Cancer Now Generations Study, a prospective UK cohort linked to the National Health Service Breast Screening Programme. Pre-diagnostic risk factors were collected at enrolment, and breast density was estimated from pre-diagnostic mammograms using Cumulus in a subset of 1,191 cases. Risk factor associations with density were estimated using linear regression models. We identified 1,185 SDBCs and 755 IBCs included in logistic regression models to estimate odds ratios (OR) and 95% confidence intervals (CI) for associations between risk factors and IBC, adjusting for age at diagnosis, time since recruitment and year of diagnosis, breast density and tumour characteristics.

**Results:**

Higher breast density was associated with later age at menarche, nulliparity, breastfeeding, history of benign breast disease (BBD), alcohol consumption, lower body mass index (BMI) at recruitment and at age 20, and current use of menopausal hormone therapy (MHT). In a fully-adjusted model, IBC risk was lower for overweight women (OR (95% CI ) = 0.74 (0.58–0.93) vs. normal weight), and higher for high breast density (2.13 (1.44–3.17) for Q4 vs. Q1), later age at menopause (1.60 (1.02–2.50) 55 + vs. < 50), current MHT use (1.41 (1.04–1.91) vs. never users), history of BBD (1.36 (1.11–1.68)), being underweight at age 20 (1.65 (1.14–2.38) vs. normal weight), family history of breast cancer (1.26 (1.00–1.58)) and ever using oral contraceptives (1.25 (0.93–1.69) vs. never). These risk factor associations were independent of tumour characteristics.

**Conclusions:**

Breast density and several risk factors independently increase the likelihood of IBC, highlighting opportunities for tailored screening strategies to enhance early detection and reduce IBCs incidence.

**Supplementary Information:**

The online version contains supplementary material available at https://doi.org/10.1186/s13058-026-02355-z.

## Introduction

The ability to detect breast cancers during routine mammographic screening (screen-detected breast cancers, SDBC), rather than in the interval between scheduled screening episodes following a negative mammogram (interval breast cancers, IBC), is a key measure for monitoring the impact of population mammographic screening programs like the UK National Health Service Breast Screening Programme (NHSBSP) [1]. This distinction of mode of detection is important because SDBCs are typically diagnosed at an earlier, more treatable stage, while IBCs are often diagnosed symptomatically, presenting with more aggressive features such as higher grade, larger size, and lymph nodal involvement [2]. Improving detection methods to reduce IBC incidence and identifying associated risk factors could inform risk-stratified screening strategies and provide important etiological insights into whether certain risk factors are likely to affect tumour development or detection. These insights can help refine our understanding of breast cancer aetiology and may support the development of more tailored screening approaches, such as adjusted screening intervals or supplemental imaging (e.g. ultrasound, magnetic resonance imaging (MRI)) for women at higher risk of IBCs.

Lower body mass index (BMI) and menopausal hormone therapy (MHT) use have been consistently found to be associated with increased chances of being diagnosed with IBC compared to SDBC [2–7]. A recent population-based cohort study in Sweden also showed strong evidence for family history of breast cancer being associated with increased risk of IBC, particularly if the affected relative also had IBC [5]. Weaker or less consistent evidence exists for other risk factors, such as reproductive factors, smoking, and benign breast disease.

Many of these factors also influence breast density [8–13], a well-established independent risk factor for breast cancer and a major determinant of mammographic sensitivity [14]. For example, MHT increases breast density, while higher BMI reduces it, with evidence for causal relationships [15], thereby affecting the sensitivity of mammographic detection. Breast density may therefore mediate or confound associations between certain risk factors and mode of detection. Adjusting for breast density is therefore essential to estimate direct and total effects, and to help disentangle underlying etiological pathways from those more likely explained by differential detectability at screening.

Moreover, several risk factors predispose to tumours with distinct characteristics, such as oestrogen receptor (ER) status, histological subtype and grade of differentiation [16–18] which may themselves affect the likelihood of detection at screening [2, 6]. Therefore, tumour characteristics could mediate or modify the relationship between risk factors and mode of detection. For example, some risk factors may predispose to faster-growing or less radiologically visible tumour types, increasing the chances of interval diagnosis. Accounting for tumour characteristics can therefore help clarify potential mechanisms underlying risk factor associations with mode of detection.

Most previous studies examining IBC risk factors have not adequately adjusted for breast density or tumour characteristics in their analyses. Many also relied on self-reported detection status without access to information on screening histories, increasing the risk of misclassification of IBCs and SDBCs [3, 4]. The Breast Cancer Now Generations Study (BGS), a large UK-based prospective cohort, offers detailed risk factor data, linkage to NHSBSP screening histories and breast tumour pathology information (https://thegenerationsstudy.co.uk/). This enabled us to examine associations between breast density, risk factors and the likelihood of IBC versus SDBC as determined from screening histories, accounting for tumour characteristics. Our analyses therefore aim to clarify the complex interplay between breast density, risk factors, detection processes, and tumour presentation.

## Methods

### Study population

**Fig. 1:**
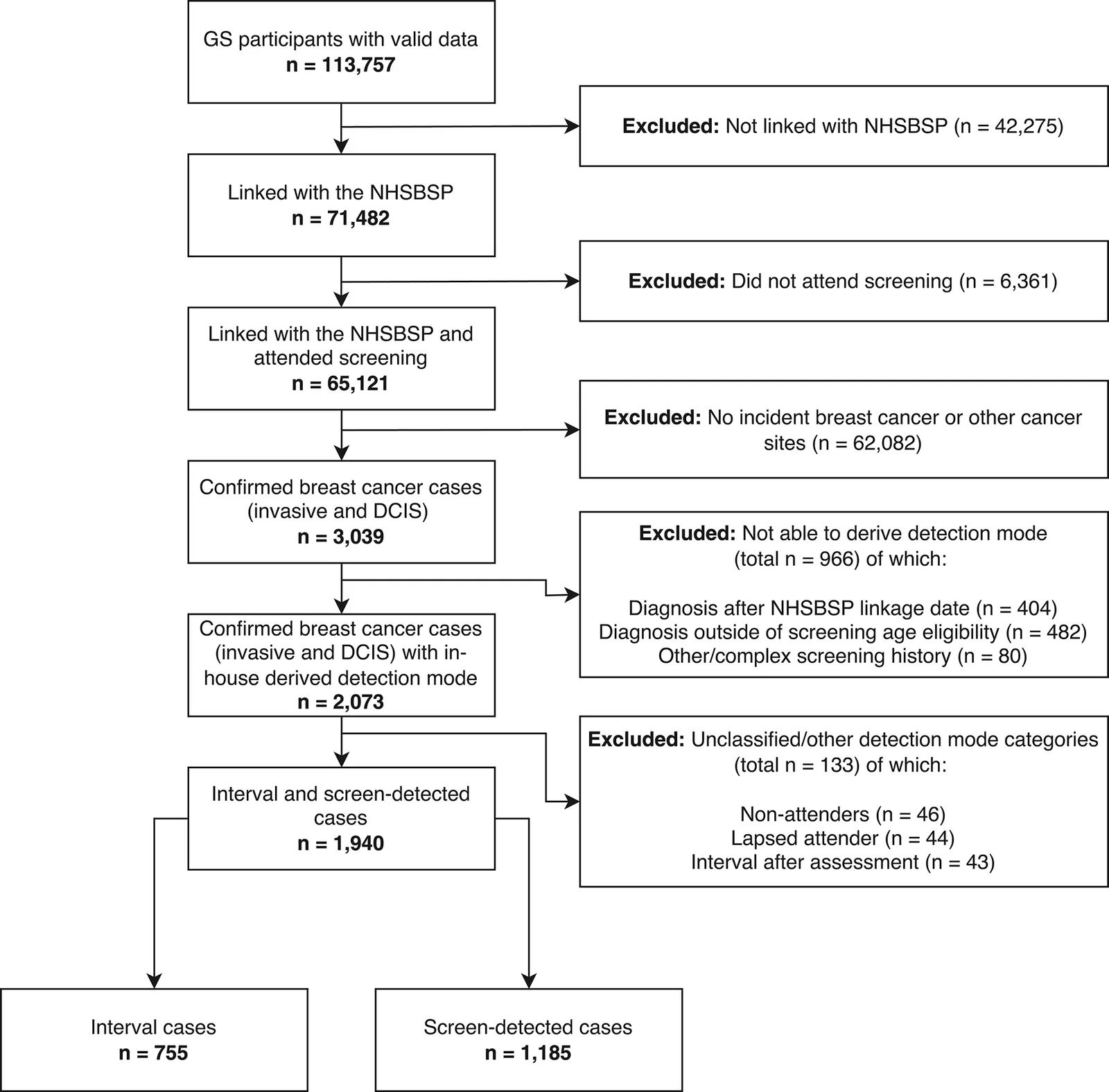
Flow chart showing the selection of generations study breast cancer cases eligible for analyses

The BGS enrolled 113,757 women between 2003 and 2021, collecting baseline data on breast cancer risk factors via questionnaires, as previously described [19]. Linkage to NHSBSP screening histories was available for 71,482 participants resident in England, of whom 65,121 (91%) attended at least one routine screen between 1988 and 2016. Among 3,039 women diagnosed with breast cancer (invasive or ductal carcinoma in situ (DCIS)) post-recruitment, 2,073 had screening records suitable for determining detection mode. Figure 1 shows the selection of BGS participants eligible for analyses.

### Breast cancer diagnosis, mode of detection and tumour characteristics

Breast cancers, invasive (International Classification of Diseases (ICD) C50) or DCIS (ICD D051, D059, D05Z), were confirmed via NHS (National Health Service) data linkages or medical records. NHSBSP linkage provided screening histories for approximately 350,000 screening episodes between 1988 and 2016. For each episode, the date of invitation, attendance and assessment and the outcome of each stage were returned. Summarised biopsy results, final action, status at episode closure and screening centre involved were supplied.

Mode of detection was derived from NHSBSP screening histories for most (78%) of cases. To maximise sample size, for women whose cancer was diagnosed after the linkage period, a screen-detected flag from cancer registration was used as a proxy. Cases were classified as SDBC if identified at or directly resulting from a routine screen, and as IBC if diagnosed following a negative screen and before the next scheduled screen. This yielded 1,185 SDBCs, 755 IBCs, and 133 unclassified cases. Tumour characteristics, including morphology, grade, size, lymph node status, and ER/PR/HER2 status, were obtained from cancer registries and pathology reports.

More details are provided in the Supplementary Methods and Supplementary Table 1.

**Table 1:** Demographic and tumour characteristics of the study population by mode of detection

Characteristic	Interval *N* = 755	Screen-detected *N* = 1,185	*p*-value^1^
*Age at recruitment, mean (SD)*	55.13 (7.11)	55.19 (6.93)	
*Age at mammogram, mean (SD)*	58.56 (5.39)	58.49 (5.37)	
Unknown, N	354	395	
*Age at diagnosis, mean (SD)*	62.32 (6.80)	61.61 (6.38)	0.306
*Time from recruitment to mammogram (years), mean (SD)*	2.54 (2.28)	2.32 (2.16)	
Unknown, N	354	395	
*Time from recruitment to diagnosis (years), mean (SD)*	7.19 (3.69)	6.42 (3.58)	0.021
*Year of diagnosis, N (%)*			
2004–2008	96 (13)	182 (15)	
2009–2013	255 (34)	496 (42)	0.256
2014–2018	404 (54)	507 (43)	0.972
*Invasive status, N (%)*			
DCIS	47 (6.2)	261 (22)	
Invasive	708 (94)	924 (78)	0.080
*Grade, N (%)*			
1	88 (12)	250 (21)	
2	335 (44)	477 (40)	0.006
3	264 (35)	187 (16)	< 0.001
Unknown, N	68	271	
*Morphology type, N (%)*			
Ductal	549 (73)	698 (59)	
Lobular	95 (13)	119 (10)	0.833
Mixed	25 (3.3)	43 (3.6)	0.777
Other	39 (5.2)	64 (5.4)	0.724
Unknown, N	47	261	
*Node status, N (%)*			
Negative	304 (40)	601 (51)	
Positive	234 (31)	194 (16)	< 0.001
Unknown, N	217	390	
*Tumour size (mm), N (%)*			
< 21	336 (45)	656 (55)	
21–50	225 (30)	162 (14)	< 0.001
50+	31 (4.1)	15 (1.3)	0.003
Unknown, N	163	352	
*ER status, N (%)*			
Positive	525 (70)	791 (67)	
Negative	137 (18)	87 (7.3)	0.007
Unknown, N	93	307	
*PR status, N (%)*			
Positive	283 (37)	375 (32)	
Negative	175 (23)	154 (13)	0.313
Unknown, N	297	656	
*HER2 status, N (%)*			
Positive	102 (14)	92 (7.8)	
Negative	524 (69)	725 (61)	0.943
Unknown, N	129	368	

^1^*p*-value from logistic regression multivariate model of all listed variables

### Questionnaire risk factors and mammographic density

Baseline recruitment questionnaires captured risk factor data including age at menarche, oral contraceptive (OC) use, parity, age at first birth, number of children, duration of breastfeeding, menopausal status, age at menopause, menopausal hormone therapy (MHT) use, alcohol units per week, body mass index (BMI) calculated from self-reported weight and heigh at age 20 and at recruitment, leisure time physical activity (in metabolic equivalents of task (MET)-hours per week), personal history of benign breast disease, and family history of breast cancer in first degree relatives. BMI was categorized according to the World Health Organisation (WHO)/UK National Institute for Health and Care Excellence (NICE) guidelines as underweight (< 18.5), normal weight (18.5–24.9), overweight (25–29.9), obese (≥ 30), not known or pregnant. For BMI at age 20, overweight and obese categories were combined due to a small number of participants.

Mammographic density was measured using Cumulus software on digital or digitised 2D mammograms (medio-lateral oblique views where available) for 1,191 cases (401 IBCs, 790 SDBCs). For all but one case we used mammograms from a screening round preceding diagnosis, and averaged density across both breasts; for one case with only a post-diagnosis mammogram available, density was measured from the contralateral, cancer-free breast (details in Supplementary Information).

### Statistical analysis

Descriptive statistics were used to summarise questionnaire-based risk factors, breast density and tumour characteristics by mode of detection (IBC or SDBC). The distribution of percent mammographic density across mode of detection and risk factor categories is shown graphically using violin plots. Differences in mean percent density were tested using linear regression models with density as the outcome variable, both unadjusted and adjusted by age at mammogram.

Logistic regression models were used to estimate odds ratios (OR) and 95% confidence intervals (CI) for the association between risk factors, breast density and tumour characteristics for IBC vs. SDBC. Covariates for inclusion in the multivariate models were selected a priori to include established risk factors for breast cancer with data available. Indicator variables for parity and menopausal status and interaction terms were included in the models to estimate ORs for risk factors defined only among parous (age at first birth, breastfeeding duration) or post-menopausal women (age at menopause, MHT status). Missing data on explanatory variables in the models was accounted by including indicator terms, allowing all participants to contribute to the analysis. We also ran sensitivity analyses restricted to women with density measurements available. To account for differential times between recruitment and breast cancer diagnosis, risk factor analyses were adjusted by age at diagnosis, time from recruitment to breast cancer diagnosis, and year of diagnosis.

We fitted four main logistic models comparing IBC with SDBC: (1) risk factors only (except breast density); (2) risk factors plus breast density, (3) risk factors plus tumour characteristics, (4) all variables. Models including breast density were further adjusted by time from mammographic density measurement to breast cancer diagnosis. This modelling strategy follows the hypothesised causal relationships among risk factors, breast density, tumour characteristics and mode of detection, which we represent as a directed acyclic graph (DAG; Supplementary Fig. 1).

Accordingly, breast density was measured on the pre-diagnostic screening mammogram and therefore precedes diagnosis, whereas tumour characteristics were ascertained at diagnosis. This temporal ordering means tumour characteristics can act only as a mediator of associations between risk factors and mode of detection, whereas breast density (intermediate in time) may act as a mediator for risk factors that causally influence density or as a confounder for risk factors merely correlated with it. The minimal sufficient adjustment set implied by the DAG for the direct effect of each risk factor on mode of detection is therefore {age/timing, breast density, tumour characteristics}, corresponding to our fully-adjusted model (Model 4); the risk-factor-only model (Model 1) estimates the total effect.

Therefore, we adjusted for breast density to account for this dual role, and for tumour characteristics to assess whether the risk factor associations with mode of detection were independent of underlying tumour biology. We present findings from models with and without adjustment for these variables to assess their respective contributions.

All statistical tests were two-sided, with an alpha-level of 0.05. Statistical analyses were performed in R version 4.4.1 (2024-06-14 ucrt) (www.rproject.org. 18). Code for statistical analyses is available on Zenodo.

## Results

Women with IBC and SDBC had similar mean ages at recruitment (~ 55 years), age at screening mammogram (~ 58 years) and age at breast cancer diagnosis (~ 62 years) (Table 1). The mean (standard deviation (SD)) time between recruitment to breast cancer diagnosis was longer for IBC (7.19 (3.69 years) than SDBC (6.42 (3.58) years) since, by definition, IBCs were diagnosed after a screening mammogram. The mean (SD) time between recruitment and density measure was much shorter since we selected pre-diagnostic mammograms to measure density, and it was similar for IBC (2.54 (2.28) years) and SDBC (2.32 (2.16) years). IBCs exhibited more aggressive characteristics than SDBCs, with higher grade, node positivity, larger size, and ER negativity being statistically significant after adjusting for all characteristics (Table 1).

**Fig. 2:**
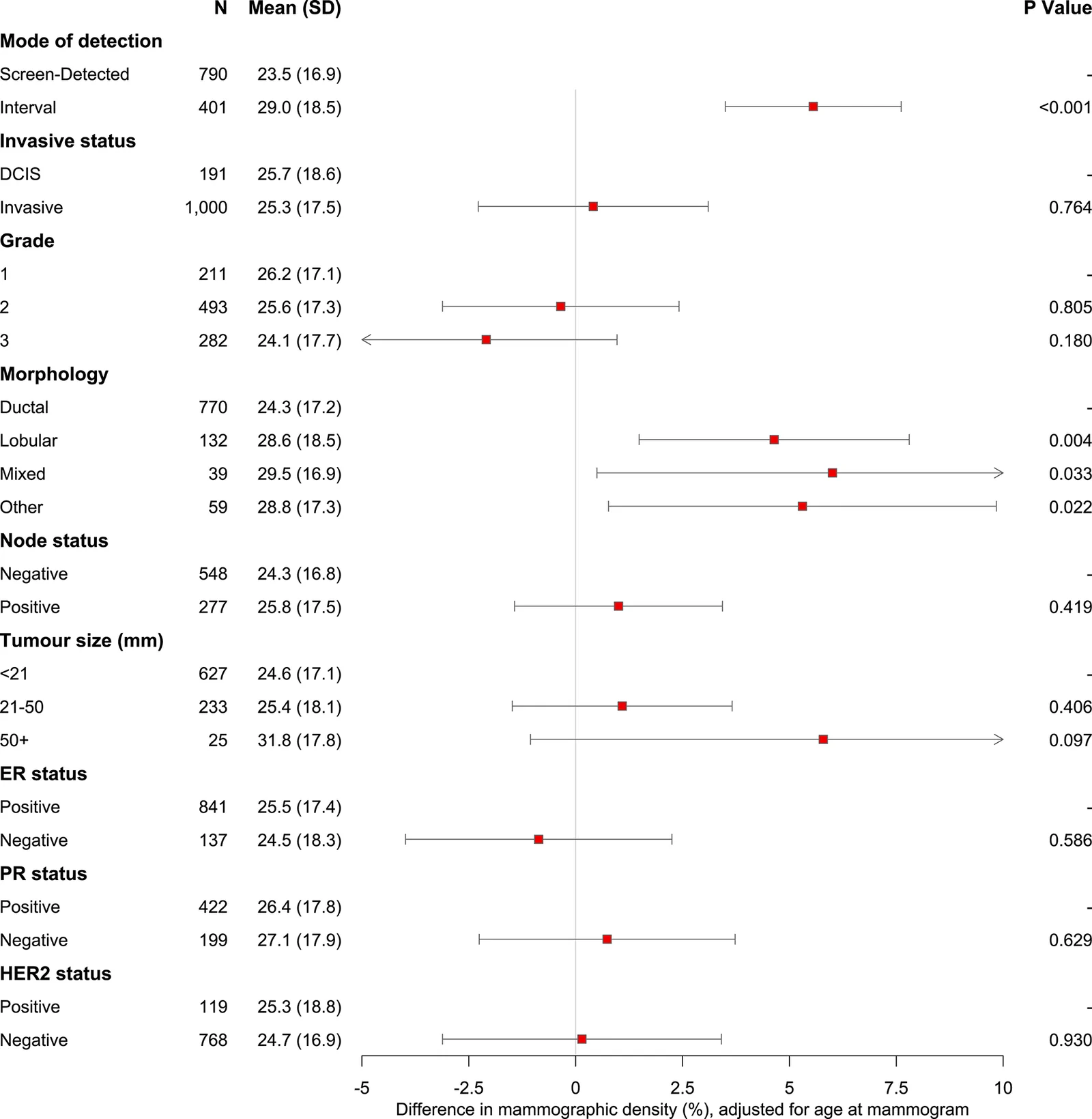
Distribution of mammographic percent density by mean and standard deviation (SD), and estimated differences with 95% confidence intervals (CI) and *P* value by mode of detection and tumour characteristics, adjusted by age at mammogram. Further details in Supplementary Table 2, including test for differences unadjusted by age at mammogram. Abbreviations: ER = Oestrogen Receptor, PR = Progesterone Receptor, HER2 = Human Epidermal growth factor Receptor 2

The mean (SD) percent density was 29% (19%) for women diagnosed with IBC and 24% (17%) for women with SDBC (Fig. 2; Supplementary Table 2; Supplementary Fig. 2). After adjusting for age at mammogram, women with IBC had, on average, 5.56% higher percent density than women with SDBC (Fig. 2; Supplementary Table 2; Supplementary Fig. 2). Respectively, women with lobular and mixed histologic types had 4.64% and 6.01% higher breast density than women with ductal tumours (Fig. 2; Supplementary Table 2; Supplementary Fig. 2). None of the other tumour characteristics were significantly associated with breast density after adjusting for age at mammogram.

**Fig. 3:**
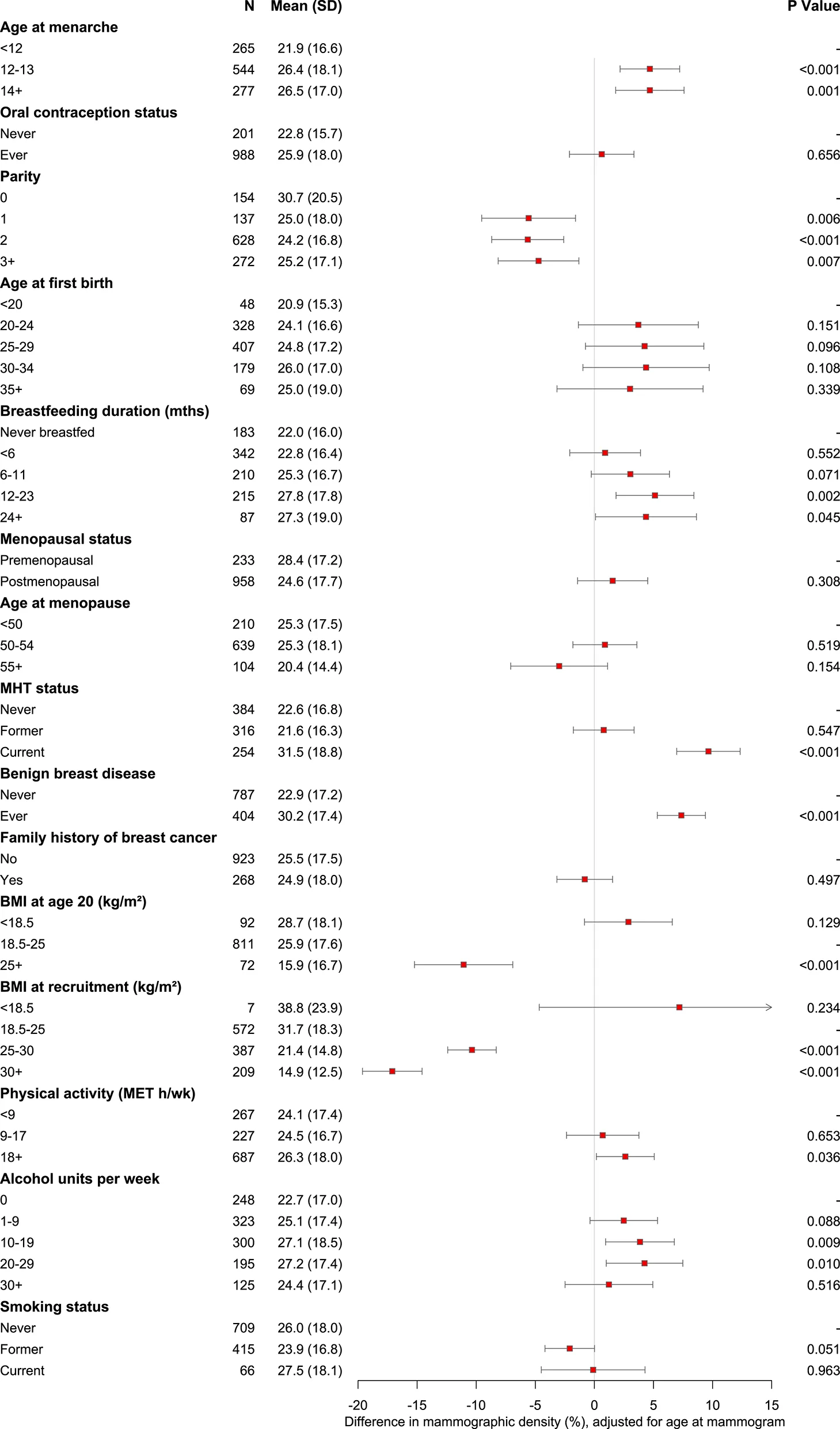
Distribution of mammographic percent density by mean and standard deviation (SD), and estimated differences with 95% confidence intervals (CI) and *P* value by risk factors for breast cancer and test for differences adjusted by age at mammogram. Further details are shown in Supplementary Table 3, including test for differences unadjusted by age at mammogram. Abbreviations: MHT = Menopausal Hormone Therapy, BMI = Body Mass Index, MET = Metabolic Equivalent of Task

In contrast, percent mammographic breast density was significantly associated with several risk factors evaluated at recruitment after adjusting by age at mammogram (Fig. 3; Supplementary Table 3; Supplementary Fig. 3). Specifically, breast density was significantly higher in participants with older age at menarche, being nulliparous, having breastfed for longer periods, currently taking MHT, having a personal history of benign breast disease (BBD), having a lower BMI at age 20 or at recruitment, physical activity, and drinking more alcohol (Fig. 3; Supplementary Table 3; Supplementary Fig. 3). The largest differences in density, after adjustment for age at mammogram, were observed for current use of MHT at recruitment (9.65% higher density than never users); obesity at recruitment (17.10% lower breast density than normal weight women); overweight/obesity at age 20 (11.07% lower density than normal weight); and BBD (7.36% higher density for presence vs. absence). Ever OC users and pre-menopausal women had higher density, but these associations were substantially attenuated and not significant after adjusting for age at mammogram. Age at first birth, age at menopause, family history of breast cancer and smoking status were not significantly associated with breast density in unadjusted or age-adjusted analyses.

Visual inspection of residual diagnostics for the above linear regression analyses using untransformed breast density revealed moderate heteroscedasticity and mild asymmetry; sensitivity analyses using square-root transformed density resolved the heteroscedasticity and yielded consistent results. Untransformed density was retained in the primary analysis to facilitate interpretation.

**Fig. 4:**
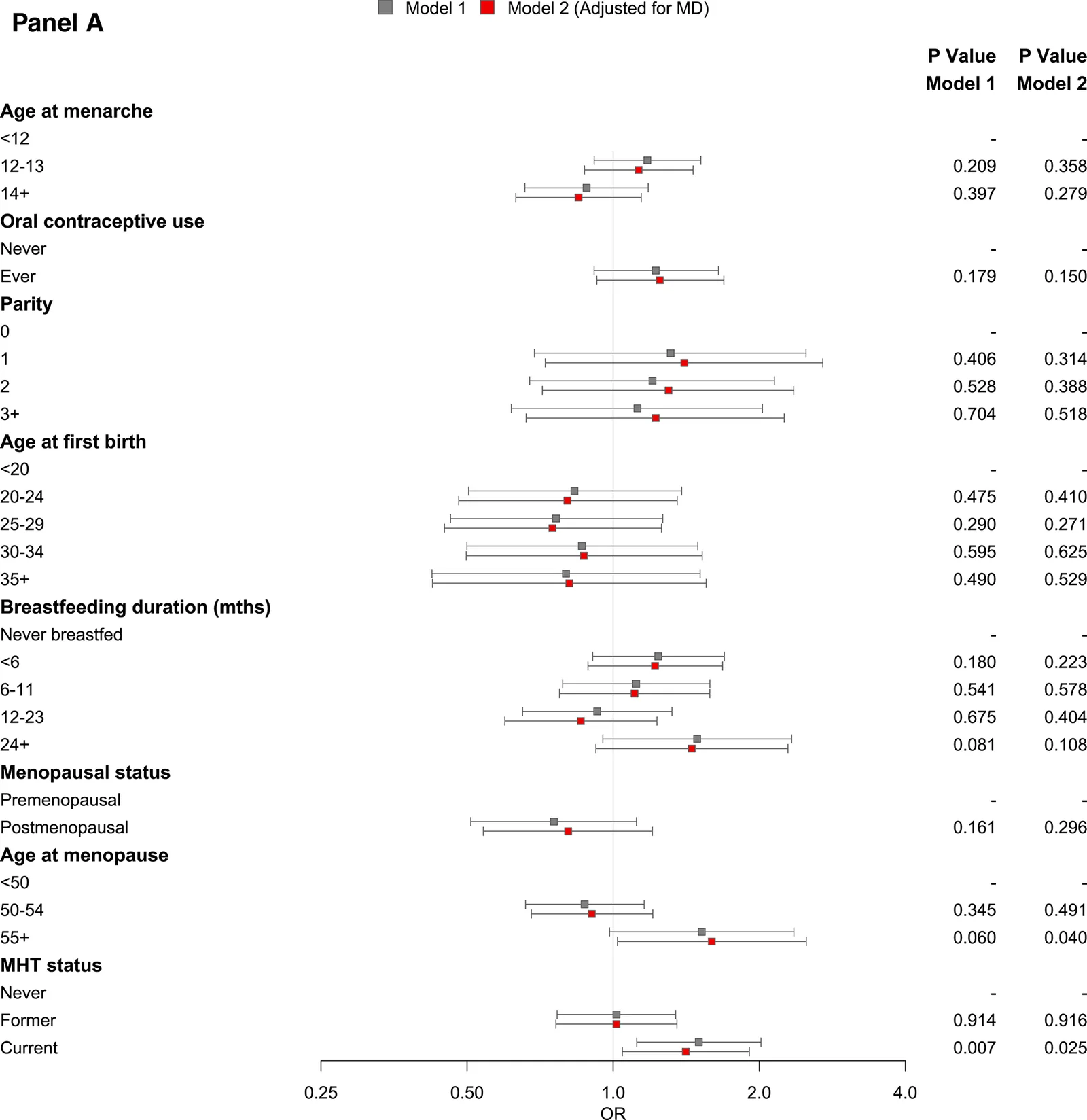

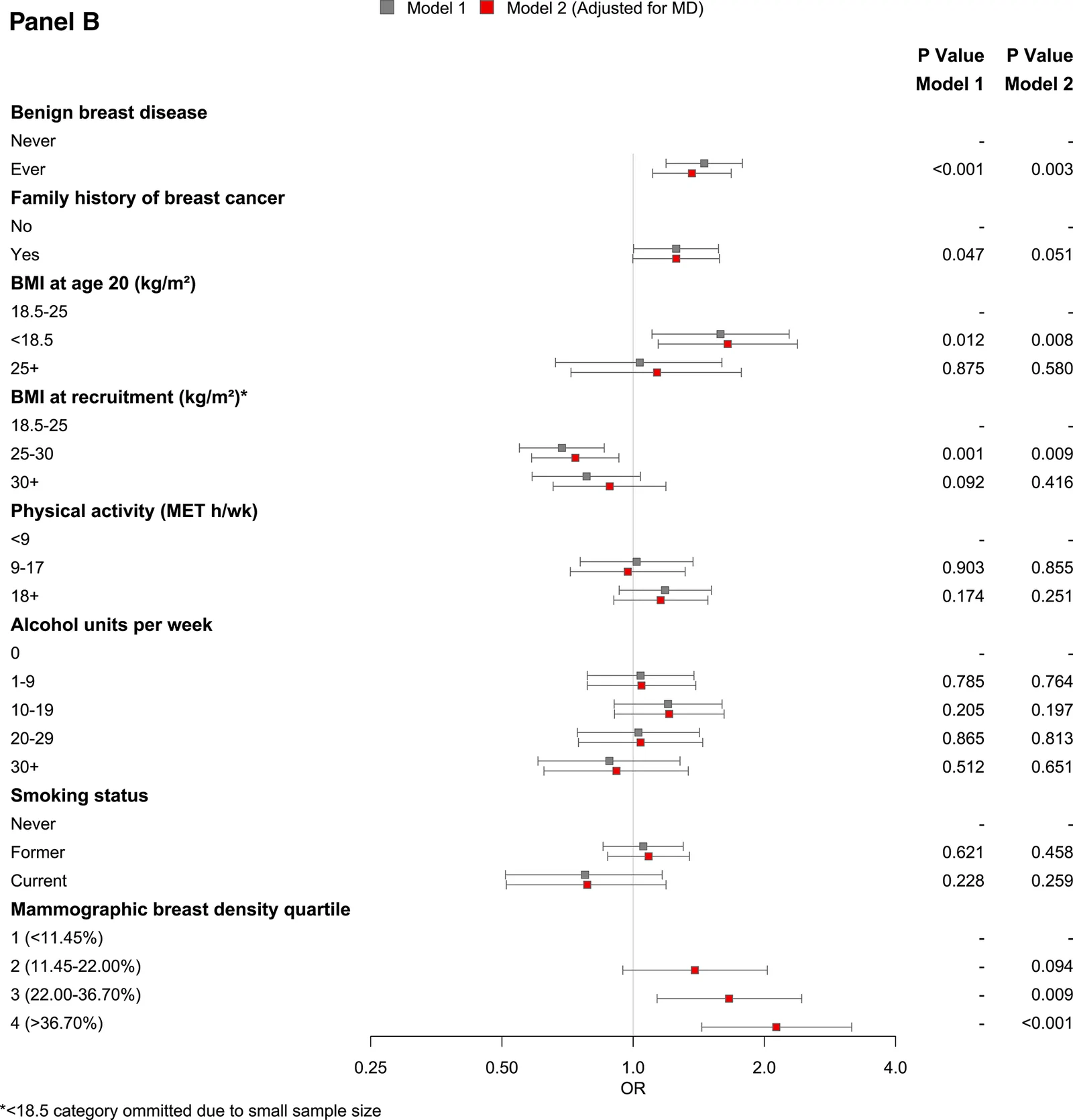
Forest plot showing the association between risk factors with interval breast cancer compared to screen-detected breast cancer, measured by the odds ratio (OR) and 95% confidence intervals (CI) from models unadjusted (model 1) and adjusted (model 2) by percent mammographic density. Panel **A** shows reproductive and hormonal factors and Panel **B** lifestyle and medical history factors. Parameter estimates are shown in Supplementary Table 4. Abbreviations: ER = Oestrogen Receptor, PR = Progesterone Receptor, HER2 = Human Epidermal growth factor Receptor 2, MHT = Menopausal Hormone Therapy, BMI = Body Mass Index, MET = Metabolic Equivalent of Task

Figure 4 and Supplementary Table 4 show estimates for the association between breast density and risk factors with mode of detection (IBC vs. SDBC) in mutually adjusted analyses. After adjusting for other risk factors, risk of IBC increased with increasing breast density (OR (95%CI) = 1.39 (0.95–2.03) for Q2 vs. Q1, 1.66 (1.14–2.44) for Q3 vs. Q1 and 2.13 (1.44–3.17) for Q4 vs. Q1). After adjusting for breast density, we identified significant associations between IBC and late age at menopause (1.60 (1.02–2.50) for 55 + vs. < 50); current use of MHT (1.41 (1.04–1.91) for current use vs. never use), and personal history of BBD (1.36 (1.11–1.68)). Although not nominally significant, women with family history of breast cancer (1.26 (1.00–1.58)) and a history of OC use (1.25 (0.93–1.69) current vs. never) were also at increased odds of IBC.

Both overweight and obese women were less likely to have a diagnosis of IBC than women with a normal weight. However, the association was stronger for overweight than obese women, and it was attenuated after adjustment for breast density. After density adjustment, ORs (95% CIs) changed from 0.69 (0.55–0.86) to 0.74 (0.58–0.93) for overweight women, and from 0.78 (0.59–1.04) to 0.88 (0.66–1.19) for obese women (Fig. 4; Supplementary Table 4). In contrast, overweight/ obesity at age 20 was not significantly associated with IBC, but women who were underweight at that age were more likely to be diagnosed with IBC compared to normal weight women (1.65 (1.14–2.38)). This association was independent of other risk factors, including their BMI at recruitment.

Sensitivity analyses restricted to women with available mammographic density data yield similar results than the main analyses including an indicator term for missingness. The risk factor associations with IBC remained after accounting for tumour characteristics (Supplementary Table 4). For cases diagnosed after the last NHSBSP linkage date, the cancer registry flag was used as proxy for derived mode of detection. Despite some discrepancies between cancer registry flags and NHSBSP-derived classifications (Supplementary Table 1), sensitivity analyses indicated that findings were robust to this definition (data not shown). Breast density was lower in digital than digitised mammograms (*p* < 0.001); however, image type was not associated with mode of detection and did not materially influence risk factors associations in sensitivity analyses (data not shown). Most cases in our study were invasive rather than DCIS (Table 1), which have distinct detectability patterns. However, a sensitivity analysis restricted to invasive breast cancers yielded similar results to the main analysis, indicating that our findings are not influenced by the inclusion of DCIS.

## Discussion

In this study of women diagnosed with breast cancer while undergoing routine mammography screening in England, we found that several established risk factors for breast cancer were associated with breast density, and that both density and risk factors independently influenced the likelihood of being diagnosed with an IBC. Specifically, late age at menopause, current use of MHT, and a history of BBD were each associated with higher likelihood of IBC, while being overweight at recruitment was associated with reduced risk. These findings build on existing evidence by simultaneously accounting for breast density and tumour characteristics- two important determinants of detectability- and by using linked screening histories rather than self-reports, thus minimizing detection and misclassification biases. Risk factor information was collected prior to breast cancer diagnosis, precluding reporting misclassification being related to mode of detection; however, this type of non-differential misclassification could have resulted in underestimation of relationships between risk factors and mode of detection. Clinically, these results may help identify women at higher risk of IBCs who could benefit from more tailored screening approaches to improve outcomes.

Multiple established risk factors for breast cancer, including older age at menarche, higher parity, longer breastfeeding duration, current MHT use, lower BMI, lower physical activity, alcohol consumption, history of BBD and family history of breast cancer, were significantly associated with higher breast density after adjustments for age at mammogram. These findings are consistent with previous reports [8–13]. For most women in our study, density was measured using the most recent pre-diagnostic mammogram, taken within a few years of the recruitment questionnaire (mean interval: 2.5 years for IBCs ,2.3 years for SDBCs). While this time gap could influence associations due to temporal changes in density or risk factors, the impact is likely small given the short interval. Our analyses also confirmed the previously reported association between higher breast density and increased chances of being diagnosed with IBC [2, 5], as well as the tendency for IBCs to present with more aggressive features, including higher grade, nodal involvement, larger size, and ER negativity [2]. These findings highlight the importance of accounting for breast density and tumour features when studying relationships between risk factors and detection mode.

Women who experience menopause at age 55 or older were 1.6 times more likely to be diagnosed with an IBC than those with menopause before age 50. Although later age at menopause was also associated with higher breast density, the association remained after adjustment. Similarly, we showed that current MHT users are 1.4 times more likely to be diagnosed with an IBC than never users, after adjusting for density, indicating a direct effect not fully mediated by density. Our findings are relevant because although the association between these menopausal factors and IBC are well established [2, 20], most reports speculated that they might be explained by their associations with a higher mammographic breast density [13]. Our results suggest that other factors not fully captured by density, such as mammographic textural features, could influence cancer detection during mammography screening [21]. Measurement error in the breast density estimates is unlikely to be related to risk factors, however it could result in residual confounding.

MHT use has been linked to lobular histology and lower-grade tumours [16, 18], both of which may affect detectability on mammography. In our data, lobular cancers were more frequent in women with dense breasts, but they occurred at similar frequencies in IBC or SDBC cases. Tumour grade was not associated with density, but IBCs were more likely to be of higher grade, consistent with faster growing being missed at screening. Thus, while grade could partially mediate the MHT association with IBC, this association remained statistically significant after adjusting for tumour grade and other tumour characteristics. Further studies are needed to clarify the mechanisms underlying the associations of late age at menopause and MHT use with IBC.

The association of IBC with a personal history of BBD is also consistent with previous reports [3, 22]. We showed that although women with BBD have higher density, it does not fully explain the association with IBC, suggesting other mechanisms. The association was independent of tumour characteristics and became stronger after accounting for these variables. As in other studies, BBD was self-reported and included a wide range of conditions such as hyperplasia, cysts, mastitis and others, which may have district mammographic features and associations with risk of IBC. For instance, calcifications have been found to be more frequent in proliferative disease with atypia, while masses are more common in lower-risk, non-proliferative BBDs [23]. These findings underscore the need for future studies to evaluate specific types of BBD and their relationship with IBC risk, as well as imaging or pathological features that may mediate this association.

Consistent with previous studies, higher BMI was associated with lower breast density and a reduced risk of IBCs [2, 4, 7, 13, 22, 24]. In our analyses, the inverse association between overweight and IBC was attenuated, although still statistically significant, after adjusting for breast density, while the association with obesity was weaker and not statistically significant before or after adjustment. These findings suggest that lower density among women with higher BMI may partly mediate the observed lower risk of IBC, likely through improved tumour visibility on mammograms. Further adjustment by socioeconomic status measured by Carstairs index, did not substantially affect this association (data not shown). If BMI is associated with delays in seeking medical care after symptoms appear, ending up being diagnosed in the next screen, this misclassification could have resulted in overestimation of the inverse association between BMI and IBC. However, we did not have information on symptoms to account for this potential bias.

These findings underscore the potential for detection bias in studies of BMI and breast cancer risk conducted in screened, predominantly post-menopausal populations. Furthermore, the findings could help explain discrepancies between observational studies, which typically show a direct association between BMI and postmenopausal breast cancer [25], and Mendelian randomization studies, which have reported inverse associations [26]. While underweight women at age 20 had similar mammographic density to normal weight women, they were more likely to be diagnosed with IBC later in life. The latter association is not consistent with a previous report [3] and warrants confirmation in future studies. Together, these findings highlight the importance of accounting for breast density and screening history when assessing associations between BMI and breast cancer risk in populations undergoing mammographic screening [27].

Family history and OC use showed weaker, statistically non-significant, associations, consistent with mixed findings in the literature. However, a recent large population-based cohort in Sweden showed that the association with IBC is stronger for those women whose relatives also had IBCs (1.18-fold increase for any breast cancer history compared to 1.70-fold increase in risk for a history of IBC), particularly when relatives were diagnosed at a younger age [5]. This suggests that more detailed family history could better identify women at higher risk of IBC. Our findings of ever OC users being more likely to be diagnosed with IBC suggest that OC use might have longer-lasting effects that are specifically relevant to IBC, which may be missed in studies examining overall breast cancer risk. The association with IBC was independent of tumour characteristics, indicating that IBC risk is not fully explained by OC use predisposing to tumour subtypes that are harder to detect on screening mammography. Previous studies on the potential associations between IBC and age at menarche, parity, age at first birth, smoking status, and alcohol consumption have been inconsistent [3–5, 7, 22] and were not confirmed in our study.

The strengths of our study include access to detailed NHSBSP screening histories, comprehensive risk factor data, breast density measurements, and detailed tumour characteristics within a prospective cohort study. These features allowed us to evaluate the impact of breast density as a confounder factor in studies of risk factors for IBC in screened populations. In addition, our analyses were restricted to women who attended routine screening, ruling out attendance bias as a confounder. This restriction ensures that our findings are directly relevant to populations undergoing mammographic screening and more accurately reflect relationships between risk factors, density, and IBC. Furthermore, we evaluated the role of tumour characteristics in detection and whether they could be potential explanations of why some risk factors predispose to tumours more likely to be diagnosed between screening rounds. Associations for key factors such as breast density, BMI and MHT appear broadly consistent across settings with shorter screening intervals [2–7], indicating the robustness of our findings; however, differences in interval length may influence the magnitude of associations and the proportion of IBCs.

This study has several limitations. Risk factors were measured at recruitment rather than at the specific time of the relevant screening, and breast density measurements were primarily taken from the screening mammogram during the cycle preceding cancer detection. This could introduce differences by mode of detection, as the time interval between the density measurement and diagnosis was, by definition, shorter for IBCs compared to SDBCs (Supplementary Fig. 4). To address these potential biases, we adjusted analyses for the time between density measurement and diagnosis, as well as the time between risk factor assessment and diagnosis. These adjustments made little difference to our estimates, suggesting that this timing is unlikely to substantially affect our results.

Although breast density was more often missing among women with IBC (47%) than SDBCs (33%), missingness was unrelated to the risk factors examined. We included a separate category for missing density in multivariable models and confirmed our results with sensitivity analyses restricted to women with density measurements. Therefore, bias in the estimated risk factor associations due to the missing-density specification is unlikely to be substantial, although it cannot be fully excluded. In addition, since we used Cumulus, a semi-automated research measure of density, translating our findings into screening practice would require alignment with measures used to quantify density in screening programs (e.g. BI-RADS categories or, increasingly, automated tools). Furthermore, availability of only a single mammogram from most (81%) of cases, precluded an assessment of longitudinal changes in density which might be biologically and clinically relevant.

Because of the lack of density data at the cohort level, we conducted case–case analyses to estimate heterogeneity in associations between risk factors and mode of detection (IBC vs. SDBC), rather than prospective cohort analyses estimating hazard ratios (HR) that directly measure the association between risk factors and breast cancer risk by mode of detection. In addition, because our analyses are restricted to breast cancer cases, they are implicitly conditioned on disease occurrence. As disease occurrence is a common effect of both risk factors and breast density, this restriction could induce spurious associations between them (collider or index-event bias; Supplementary Fig. 1). However, the consistency of findings with previous reports suggests that this bias is unlikely to substantially influence our main conclusions. Furthermore, adjustment for breast density mitigates this potential bias in the association between risk factors and mode of detection.

Additionally, the study lacked access to radiological reviews, which would have enabled differentiation between false-negative, occult or minimal-sign IBCs (missed cancers) and true interval cancers. Occult IBCs are strongly associated with high breast density, where tumours are more challenging to detect radiologically, whereas the density association is weaker for true interval cancers or false negatives [6]. Using proxy measures for true IBC (e.g. those in women with low breast density) or incorporating radiological reviews in future studies could provide further insights into the interplay between density, detection biases, and tumour biology.

## Conclusions

Our findings, together with previous literature, provide strong evidence that breast density and several risk factors independently predict IBC risk. Understanding how these factors influence risk and tumour detectability could help distinguish true aetiological associations from detection bias, and inform risk stratification strategies, such as adjusted intervals or supplementary imaging, ultimately improving screening outcomes.

## Supplementary Information

Below is the link to the electronic supplementary material.

Supplementary Material 1

## Data Availability

The data underlying this study are from the Breast Cancer Now Generations Study and are not publicly available due to participant confidentiality and consent restrictions. De-identified data can be requested for approved research through the study’s controlled access process. Information on how to apply for access is available at https://thegenerationsstudy.co.uk/.

## Abbreviations

BBD: Benign breast disease
BGS: Breast Cancer Now Generations Study
BI-RADS: Breast Imaging Reporting Data System
BMI: Body mass index
CI: Confidence interval
DCIS: Ductal carcinoma in–situ
ER: Oestrogen receptor
HER2: Human epidermal growth factor receptor 2
IBC: Interval breast cancer
ICD: International classification of diseases
MET: Metabolic equivalent of task
MHT: Menopausal hormone therapy
MRI: Magnetic resonance imaging
NICE: National institute for health and care excellence
NHS: National health service
NHSBSP: National health service breast screening programme
OC: Oral contraceptives
OR: Odds ratio
PR: Progesterone receptor
SDBC: Screen–detected breast cancer
WHO: World Health Organisation
