# Supplementary material for: Breast density and risk factors for interval and screen-detected breast cancers: a case–case analysis of the Breast Cancer Now Generations Study, UK
Source: Breast Cancer Res. 2026 Aug 13;28:191. doi: 10.1186/s13058-026-02355-z (PMC13753370; doi:10.1186/s13058-026-02355-z)
Supplement: Supplementary file 1 [file 13058_2026_2355_MOESM1_ESM.docx]

**SUPPLEMENTARY INFORMATION**

**Breast cancer tumour characteristics and screening data**

Breast cancers were defined as incident ductal carcinoma *in situ* (DCIS; ICD code D051 and unspecified codes D059 or D05Z assumed to be DCIS (N=3)) or invasive breast cancer (ICD codes starting with C50). Information on tumour characteristics (morphology, histological grade, tumour size, lymph node involvement, oestrogen receptor (ER), progesterone receptor (PR) and human epidermal growth factor receptor 2 (HER2) status were obtained from cancer registrations and pathology reports.

The National Health Service Breast Screening Programme (NHSBSP) in England is a nationally organised programme that operates through around 80 breast screening facilities and offers routine screening to eligible women aged 50-70 in 3-year intervals. Linkage between the BGS cohort and NHSBSP data was performed between January and May 2016. Women eligible for linkage were born before 1977, had a valid NHS number and had provided the relevant consent. Linkage resulted in breast screening histories for 71,482 women residents in England with approximately 350,000 screening episodes between 1988-2016. For each episode, the date of invitation, attendance and assessment and the outcome of each stage were returned. Summarised biopsy results, final action and status at episode closure were also supplied in addition to the screening centre where the episode took place. When available, for cases diagnosed after the latest episode information supplied by NHSBSP linkage, we used the screen-detected flag from the cancer registration and assumed that cancers not flagged as screen-detected were interval cancers (**Supplementary Table 1**).

**Derivation of mode of detection during mammographic screening**

Mode of detection was derived using NHSBSP screening histories. Cases were designated as screen-detected breast cancer (SDBC) if they were detected at, or directly resulting from, findings at a routine screen, using NHSBSP screening histories. This includes cases diagnosed as a result of assessment following an abnormal screen, and cases diagnosed at an early recall screen that was prompted by the findings of a routine screen.

Cases were designated as interval breast cancer (IBC) if they were detected and diagnosed following a negative (normal) routine screen (and associated assessment where relevant) and before the next scheduled screen. For women whose screening episode occurred within the available data linkage period, but with cancer diagnosis after the linkage period, we identified diagnoses between 3 to 36 months after an attended negative routine screening episode as IBC.

An individual must have attended at least one routine screen for their breast cancer to qualify as SDBC or IBC. Not all breast cancers diagnosed in screening attenders fell into these two categories. For example, a woman may attend a screen then chooses not to attend her next invitation. If she is subsequently diagnosed with breast cancers, she is neither an IBC nor an SDBC but is designated a lapsed attender. She may self-refer and have her breast cancer detected, but the self-referral may have been prompted by symptoms and cannot be considered screen detected.

For most cases (86%), mode of detection was derived using the date of breast cancer diagnosis in conjunction with the detailed screening histories obtained from NHSBSP linkage. For women diagnosed after the last linkage date, a screen-detected flag provided by cancer registration was used as a proxy for SDBC (screen-detected flag = Yes) and IBC (screen-detected flag = No). **Supplementary Table 1** shows the cross-classification of the cancer registration screen-detected flag and our derived mode of detection variable (SDBC vs IBC) derived using screening history data. For women with data from both sources, 85% (852 out of 1004) of those with screen-detected flag = yes were identified as SBDC and 56% (501 out of 898) of cases with flag = no were identified as IBC. This indicates that while this flag is a reasonable proxy for SDBC, identification of IBC is less accurate since the “not screen detected” category includes a substantial number of women with other detection routes.

An R-coded algorithm was developed to derived mode of detection from the detailed screening history, augmented by the cancer registry screen-detected flag where appropriate. For a minority of complex cases (7%), the mode of detection was set using expert review of the screening histories. For example, if a woman moved screening centre and was not reinvited three years after their previous invitation, a self-referral or a referral for screening by their GP around her 3-year anniversary was considered routine. Such decisions were only made possible due to the detailed nature of the NHSBSP episode data available.

According to our detection mode derived for cases diagnosed pre-linkage, 99% of SDBC are diagnosed within 3 months of last routine screen and 98% of IBC after ≥ 3 months. We are using an upper limit of 36 months due to the possibility of subsequent routine screens that we are yet unaware of.

*Derivation of mode of detection for breast cancer cases diagnosed post-linkage*

The linkage date was not a single date but varied by screening centre. Centre-specific linkage dates were determined from the maximum date within closed NHSBSP episodes for each centre. For a breast cancer diagnosed after the relevant linkage date but within 36 months of the last routine episode attended, the following derivation rules were applied: if the date of diagnosis was <3months after the last routine screen attended, and the screen resulted in positive endpoints leading to an assessment, the case was categorised as SDBC. If the date of diagnosis was 3-36 months after the last routine screen attended with negative endpoint (normal), the case was categorised as IBC. For a breast cancer diagnosed after the relevant linkage date without a routine episode attendance within 36 months (13.7%), the following derivation rules were applied: if the woman was within screening invitation age at diagnosis and cancer registry screen detected variable was “yes”: the case was designated SDBC. If “no” and with age diagnosis at 76 years or less, the case was considered IBC. Otherwise, the detection mode remained unknown. The age of 76 was chosen as three years after the expected age at last screen. Age at last screen was set to 73 since many centres took part in [AgeX trial](https://www.cancerresearchuk.org/about-cancer/find-a-clinical-trial/a-study-to-evaluate-an-age-extension-of-the-nhs-breast-screening-programme#undefined) to evaluate an age extension to the NHS Breast Screening Programme that extended the end of screening age from 70 to 73 years.

The relevant screening date was defined as the screen prior to the screen at which the cancer was detected (diagnostic screen) for SDBC, and the last negative screen prior to diagnosis for IBC. Derivation of relevant screen date for cases diagnosed post-linkage was estimated based on median time interval between relevant screen and diagnosis date for SDBC and IBC with known relevant screen, 22 days and 22 months, respectively.

**Mammographic Density Assessment**

Mammographic density assessment methods are described in Schoemaker et al. [15]. Briefly, analogue and digital mammographic images were obtained from NHSBSP screening centres, and percentage density was estimated using Cumulus software (Sunnybrook Health Sciences Centre, Toronto, Canada). Density readings were taken from the most recent pre-diagnostic mammogram, averaged across both breasts. For SDBCs, this was the negative screen prior to the one detecting cancer; for IBCs, it was the last negative screen before diagnosis. If this was unavailable, readings were taken from the contralateral breast or an earlier screen where possible. **Supplementary Figure 4** shows a graphical representation of different scenarios on the timing of recruitment when risk factors were assessed, breast density assessment and breast cancer diagnosis/detection, in relation to routine screening rounds occurring every three years. Medio-lateral oblique (MLO) views were preferred; cranio-caudal views were used when MLO was unavailable. Mammographic density readings were available for 1,191 women, including 401 IBCs (53% of total) and 790 SDBCs (67% of total). Of the 1,191 density readings selected for analysis, 1,190 were pre-diagnostic, with one contralateral post-diagnosis density reading being included in analysis. Missing data was mostly due to lack of availability of mammograms from the screening centres for cancers diagnosed many years ago.

**Supplementary Table 1:** Cross-classification of screen-detection mode derived from the National Breast Screening Programme (NBCSP) screening histories, and the cancer registry screen-detected flag. Table only includes women with NBCSP-derived detection mode available, and where the registry screen-detected flag was not used to define detection mode.

|  | **NBCSP-Derived Detection Mode** | | | |
| --- | --- | --- | --- | --- |
| **Registry Detection Mode** | Interval | Screen-Detected | Other | **Total** |
| Interval | 501 | 0 | 397 | 898 (34%) |
| Screen-Detected | 33 | 852 | 119 | 1,004 (38%) |
| Unknown | 17 | 6 | 56 | 79 (3.0%) |
| No Data | 62 | 50 | 522 | 634 (24%) |
| **Total** | 613 (23%) | 908 (35%) | 1,094 (42%) | 2,615 (100%) |

**Supplementary Table 2:** Univariable association between percent mammographic density with mode of detection and tumour characteristics both unadjusted and adjusted by age at mammogram. Corresponding 95% confidence intervals (CI) and P values also presented. Abbreviations: ER = Oestrogen Receptor, PR = Progesterone Receptor, HER2 = Human Epidermal growth factor Receptor 2.

|  | | **Density** | **Unadjusted** | | | **Adjusted for age at mammogram** | | |
| --- | --- | --- | --- | --- | --- | --- | --- | --- |
| **Characteristic** | **N = 1,191**^1^ | **N = 1,191**^2^ | **Beta** | **95% CI**^3^ | **p-value** | **Beta** | **95% CI**^3^ | **p-value** |
| **Mode of detection** |  |  |  |  |  |  |  |  |
| Screen-Detected | 790 | 23.52 (16.86) | — | — |  | — | — |  |
| Interval | 401 | 29.03 (18.55) | 5.51 | 3.41, 7.61 | <0.001 | 5.56 | 3.50, 7.61 | <0.001 |
| **Invasive status** |  |  |  |  |  |  |  |  |
| DCIS | 191 | 25.70 (18.58) | — | — |  | — | — |  |
| Invasive | 1,000 | 25.32 (17.46) | -0.39 | -3.12, 2.35 | 0.782 | 0.41 | -2.28, 3.10 | 0.764 |
| **Grade** |  |  |  |  |  |  |  |  |
| 1 | 211 | 26.18 (17.13) | — | — |  | — | — |  |
| 2 | 493 | 25.63 (17.30) | -0.55 | -3.36, 2.26 | 0.701 | -0.35 | -3.12, 2.42 | 0.805 |
| 3 | 282 | 24.10 (17.75) | -2.08 | -5.19, 1.03 | 0.189 | -2.09 | -5.15, 0.97 | 0.180 |
| **Morphology** |  |  |  |  |  |  |  |  |
| Ductal | 770 | 24.27 (17.21) | — | — |  | — | — |  |
| Lobular | 132 | 28.64 (18.45) | 4.37 | 1.16, 7.58 | 0.008 | 4.64 | 1.48, 7.80 | 0.004 |
| Mixed | 39 | 29.53 (16.85) | 5.26 | -0.34, 10.86 | 0.065 | 6.01 | 0.50, 11.52 | 0.033 |
| Other | 59 | 28.79 (17.32) | 4.52 | -0.08, 9.13 | 0.054 | 5.30 | 0.77, 9.84 | 0.022 |
| **Node status** |  |  |  |  |  |  |  |  |
| Negative | 548 | 24.30 (16.81) | — | — |  | — | — |  |
| Positive | 277 | 25.75 (17.49) | 1.45 | -1.02, 3.92 | 0.248 | 1.00 | -1.43, 3.43 | 0.419 |
| **Tumour size (mm)** |  |  |  |  |  |  |  |  |
| <21 | 627 | 24.56 (17.12) | — | — |  | — | — |  |
| 21-50 | 233 | 25.43 (18.06) | 0.86 | -1.75, 3.48 | 0.517 | 1.09 | -1.48, 3.66 | 0.406 |
| 50+ | 25 | 31.83 (17.76) | 7.27 | 0.31, 14.23 | 0.041 | 5.79 | -1.06, 12.64 | 0.097 |
| **ER status** |  |  |  |  |  |  |  |  |
| Positive | 841 | 25.47 (17.37) | — | — |  | — | — |  |
| Negative | 137 | 24.46 (18.25) | -1.01 | -4.18, 2.15 | 0.530 | -0.86 | -3.98, 2.25 | 0.586 |
| **PR status** |  |  |  |  |  |  |  |  |
| Positive | 422 | 26.41 (17.77) | — | — |  | — | — |  |
| Negative | 199 | 27.08 (17.92) | 0.67 | -2.33, 3.68 | 0.660 | 0.74 | -2.26, 3.73 | 0.629 |
| **HER2 status** |  |  |  |  |  |  |  |  |
| Positive | 119 | 25.30 (18.78) | — | — |  | — | — |  |
| Negative | 768 | 24.73 (16.92) | -0.56 | -3.88, 2.76 | 0.740 | 0.15 | -3.12, 3.41 | 0.930 |
| ^1^n | | | | | | | | |
| ^2^Mean (SD) | | | | | | | | |
| ^3^CI = Confidence Interval | | | | | | | | |

**Supplementary Table 3:** Univariable association between percent mammographic density with risk factors both unadjusted and adjusted by age at mammogram. Corresponding 95% confidence intervals (CI) and P values also presented. Abbreviations: MHT = Menopausal Hormone Therapy, BMI = Body Mass Index, MET = Metabolic Equivalent of Task.

|  | | **Density** | **Unadjusted** | | | **Adjusted for age at mammogram** | | |
| --- | --- | --- | --- | --- | --- | --- | --- | --- |
| **Characteristic** | **N = 1,191**^1^ | **N = 1,191**^2^ | **Beta** | **95% CI**^3^ | **p-value** | **Beta** | **95% CI**^3^ | **p-value** |
| **Age at menarche** |  |  |  |  |  |  |  |  |
| <12 | 265 | 21.90 (16.61) | — | — |  | — | — |  |
| 12-13 | 544 | 26.43 (18.09) | 4.53 | 1.96, 7.09 | <0.001 | 4.69 | 2.17, 7.22 | <0.001 |
| 14+ | 277 | 26.52 (17.01) | 4.62 | 1.67, 7.56 | 0.002 | 4.69 | 1.80, 7.59 | 0.001 |
| **Oral contraception status** |  |  |  |  |  |  |  |  |
| Never | 201 | 22.79 (15.69) | — | — |  | — | — |  |
| Ever | 988 | 25.93 (17.96) | 3.14 | 0.47, 5.82 | 0.021 | 0.62 | -2.11, 3.35 | 0.656 |
| **Parity** |  |  |  |  |  |  |  |  |
| 0 | 154 | 30.70 (20.53) | — | — |  | — | — |  |
| 1 | 137 | 25.03 (18.04) | -5.67 | -9.71, -1.63 | 0.006 | -5.57 | -9.53, -1.60 | 0.006 |
| 2 | 628 | 24.22 (16.77) | -6.49 | -9.58, -3.39 | <0.001 | -5.64 | -8.69, -2.59 | <0.001 |
| 3+ | 272 | 25.22 (17.15) | -5.48 | -8.95, -2.01 | 0.002 | -4.73 | -8.14, -1.31 | 0.007 |
| **Age at first birth** |  |  |  |  |  |  |  |  |
| <20 | 48 | 20.86 (15.30) | — | — |  | — | — |  |
| 20-24 | 328 | 24.08 (16.58) | 3.22 | -1.94, 8.38 | 0.221 | 3.72 | -1.36, 8.79 | 0.151 |
| 25-29 | 407 | 24.75 (17.19) | 3.89 | -1.20, 8.99 | 0.134 | 4.25 | -0.76, 9.26 | 0.096 |
| 30-34 | 179 | 25.97 (17.04) | 5.11 | -0.31, 10.54 | 0.065 | 4.37 | -0.97, 9.71 | 0.108 |
| 35+ | 69 | 25.02 (18.96) | 4.16 | -2.12, 10.43 | 0.194 | 3.02 | -3.16, 9.19 | 0.339 |
| **Breastfeeding duration (mths)** |  |  |  |  |  |  |  |  |
| Never breastfed | 183 | 21.96 (15.98) | — | — |  | — | — |  |
| <6 | 342 | 22.84 (16.41) | 0.88 | -2.16, 3.92 | 0.569 | 0.91 | -2.09, 3.90 | 0.552 |
| 6-11 | 210 | 25.32 (16.68) | 3.36 | 0.00, 6.71 | 0.050 | 3.04 | -0.26, 6.35 | 0.071 |
| 12-23 | 215 | 27.79 (17.78) | 5.83 | 2.49, 9.17 | <0.001 | 5.12 | 1.83, 8.42 | 0.002 |
| 24+ | 87 | 27.29 (19.01) | 5.33 | 1.01, 9.65 | 0.016 | 4.37 | 0.10, 8.63 | 0.045 |
| **Menopausal status** |  |  |  |  |  |  |  |  |
| Premenopausal | 233 | 28.39 (17.23) | — | — |  | — | — |  |
| Postmenopausal | 958 | 24.65 (17.66) | -3.74 | -6.26, -1.22 | 0.004 | 1.54 | -1.43, 4.51 | 0.308 |
| **Age at menopause** |  |  |  |  |  |  |  |  |
| <50 | 210 | 25.28 (17.53) | — | — |  | — | — |  |
| 50-54 | 639 | 25.25 (18.11) | -0.02 | -2.77, 2.73 | 0.986 | 0.89 | -1.82, 3.60 | 0.519 |
| 55+ | 104 | 20.39 (14.38) | -4.89 | -9.04, -0.75 | 0.021 | -2.98 | -7.08, 1.12 | 0.154 |
| **MHT status** |  |  |  |  |  |  |  |  |
| Never | 384 | 22.65 (16.83) | — | — |  | — | — |  |
| Former | 316 | 21.57 (16.28) | -1.08 | -3.64, 1.49 | 0.411 | 0.79 | -1.78, 3.36 | 0.547 |
| Current | 254 | 31.52 (18.78) | 8.87 | 6.14, 11.60 | <0.001 | 9.65 | 6.97, 12.33 | <0.001 |
| **Benign breast disease** |  |  |  |  |  |  |  |  |
| Never | 787 | 22.93 (17.23) | — | — |  | — | — |  |
| Ever | 404 | 30.15 (17.45) | 7.23 | 5.15, 9.31 | <0.001 | 7.36 | 5.32, 9.39 | <0.001 |
| **Family history of breast cancer** |  |  |  |  |  |  |  |  |
| No | 923 | 25.50 (17.54) | — | — |  | — | — |  |
| Yes | 268 | 24.94 (17.96) | -0.56 | -2.96, 1.84 | 0.645 | -0.82 | -3.17, 1.54 | 0.497 |
| **BMI at age 20 (kg/m²)** |  |  |  |  |  |  |  |  |
| <18.5 | 92 | 28.73 (18.05) | 2.86 | -0.93, 6.65 | 0.138 | 2.88 | -0.83, 6.59 | 0.129 |
| 18.5-25 | 811 | 25.87 (17.57) | — | — |  | — | — |  |
| 25+ | 72 | 15.89 (16.65) | -9.98 | -14.22, -5.75 | <0.001 | -11.07 | -15.23, -6.91 | <0.001 |
| **BMI at recruitment (kg/m**²**)** |  |  |  |  |  |  |  |  |
| <18.5 | 7 | 38.84 (23.95) | 7.11 | -5.04, 19.27 | 0.251 | 7.19 | -4.67, 19.05 | 0.234 |
| 18.5-25 | 572 | 31.73 (18.26) | — | — |  | — | — |  |
| 25-30 | 387 | 21.38 (14.79) | -10.35 | -12.46, -8.25 | <0.001 | -10.36 | -12.41, -8.31 | <0.001 |
| 30+ | 209 | 14.88 (12.53) | -16.85 | -19.43, -14.27 | <0.001 | -17.10 | -19.62, -14.58 | <0.001 |
| **Physical activity (MET h/wk)** |  |  |  |  |  |  |  |  |
| <9 | 267 | 24.09 (17.45) | — | — |  | — | — |  |
| 9-17 | 227 | 24.54 (16.69) | 0.44 | -2.68, 3.57 | 0.781 | 0.70 | -2.36, 3.77 | 0.653 |
| 18+ | 687 | 26.28 (18.02) | 2.18 | -0.31, 4.68 | 0.086 | 2.62 | 0.17, 5.06 | 0.036 |
| **Alcohol units per week** |  |  |  |  |  |  |  |  |
| 0 | 248 | 22.74 (17.03) | — | — |  | — | — |  |
| 1-9 | 323 | 25.14 (17.40) | 2.40 | -0.51, 5.31 | 0.106 | 2.48 | -0.37, 5.34 | 0.088 |
| 10-19 | 300 | 27.06 (18.55) | 4.32 | 1.35, 7.28 | 0.004 | 3.86 | 0.95, 6.77 | 0.009 |
| 20-29 | 195 | 27.19 (17.38) | 4.45 | 1.15, 7.75 | 0.008 | 4.24 | 1.00, 7.48 | 0.010 |
| 30+ | 125 | 24.37 (17.06) | 1.63 | -2.16, 5.41 | 0.399 | 1.23 | -2.48, 4.95 | 0.516 |
| **Smoking status** |  |  |  |  |  |  |  |  |
| Never | 709 | 26.05 (18.02) | — | — |  | — | — |  |
| Former | 415 | 23.88 (16.83) | -2.17 | -4.30, -0.03 | 0.047 | -2.09 | -4.18, 0.01 | 0.051 |
| Current | 66 | 27.52 (18.11) | 1.47 | -2.97, 5.92 | 0.516 | -0.10 | -4.49, 4.28 | 0.963 |
| ^1^n | | | | | | | | |
| ^2^Mean (SD) | | | | | | | | |
| ^3^CI = Confidence Interval | | | | | | | | |

**Supplementary Table 4:** Association of tumour characteristics and risk factors with interval breast cancer compared to screen-detected breast cancer measure by odds ratios (OR) and 95% confidence intervals (CIs) estimated from four logistic regression models: model 1 included only risk factors as explanatory variables; model 2 included risk factors and mammographic density; model 3 included risk factors and tumour characteristics; model 4 included all variables included in model 3, as well as mammographic density. Abbreviations: ER = Oestrogen Receptor, PR = Progesterone Receptor, HER2 = Human Epidermal growth factor Receptor 2, MHT = Menopausal Hormone Therapy, BMI = Body Mass Index, MET = Metabolic Equivalent of Task.

|  | **Distribution by detection mode** | | **Model 1**^4^ | | | **Model 2**^5^ | | | **Model 3**^6^ | | | **Model 4**^7^ | | |
| --- | --- | --- | --- | --- | --- | --- | --- | --- | --- | --- | --- | --- | --- | --- |
| **Characteristic** | **Interval**  N = 755^1^ | **Screen-Detected**  N = 1,185^1^ | **OR**^2^ | **95% CI**^3^ | **p-value** | **OR**^2^ | **95% CI**^3^ | **p-value** | **OR**^2^ | **95% CI**^3^ | **p-value** | **OR**^2^ | **95% CI**^3^ | **p-value** |
| **Age at menarche** |  |  |  |  |  |  |  |  |  |  |  |  |  |  |
| <12 | 153 (20) | 260 (22) | — | — |  | — | — |  | — | — |  | — | — |  |
| 12-13 | 369 (49) | 523 (44) | 1.18 | 0.91, 1.52 | 0.209 | 1.13 | 0.87, 1.46 | 0.358 | 1.20 | 0.91, 1.58 | 0.203 | 1.14 | 0.86, 1.51 | 0.376 |
| 14+ | 169 (22) | 286 (24) | 0.88 | 0.66, 1.18 | 0.397 | 0.85 | 0.63, 1.14 | 0.279 | 0.93 | 0.67, 1.28 | 0.648 | 0.88 | 0.63, 1.21 | 0.424 |
| Missing | 64 (8.5) | 116 (9.8) |  |  |  |  |  |  |  |  |  |  |  |  |
| **Oral contraceptive use** |  |  |  |  |  |  |  |  |  |  |  |  |  |  |
| Never | 95 (13) | 182 (15) | — | — |  | — | — |  | — | — |  | — | — |  |
| Ever | 659 (87) | 1,001 (84) | 1.22 | 0.91, 1.65 | 0.179 | 1.25 | 0.93, 1.69 | 0.150 | 1.30 | 0.95, 1.80 | 0.109 | 1.32 | 0.95, 1.84 | 0.096 |
| Missing | 1 (0.1) | 2 (0.2) |  |  |  |  |  |  |  |  |  |  |  |  |
| **Parity** |  |  |  |  |  |  |  |  |  |  |  |  |  |  |
| 0 | 97 (13) | 161 (14) | — | — |  | — | — |  | — | — |  | — | — |  |
| 1 | 86 (11) | 125 (11) | 1.31 | 0.69, 2.50 | 0.406 | 1.40 | 0.72, 2.70 | 0.314 | 1.12 | 0.55, 2.26 | 0.752 | 1.14 | 0.55, 2.33 | 0.722 |
| 2 | 408 (54) | 630 (53) | 1.21 | 0.67, 2.15 | 0.528 | 1.30 | 0.71, 2.36 | 0.388 | 0.98 | 0.52, 1.84 | 0.941 | 1.02 | 0.53, 1.95 | 0.941 |
| 3+ | 164 (22) | 269 (23) | 1.12 | 0.62, 2.03 | 0.704 | 1.22 | 0.66, 2.25 | 0.518 | 0.98 | 0.51, 1.87 | 0.943 | 1.02 | 0.52, 1.99 | 0.942 |
| **Age at first birth** |  |  |  |  |  |  |  |  |  |  |  |  |  |  |
| <20 | 35 (4.6) | 45 (3.8) | — | — |  | — | — |  | — | — |  | — | — |  |
| 20-24 | 197 (26) | 308 (26) | 0.83 | 0.50, 1.38 | 0.475 | 0.80 | 0.48, 1.35 | 0.410 | 0.77 | 0.45, 1.34 | 0.354 | 0.76 | 0.44, 1.33 | 0.334 |
| 25-29 | 256 (34) | 421 (36) | 0.76 | 0.46, 1.27 | 0.290 | 0.75 | 0.45, 1.26 | 0.271 | 0.74 | 0.43, 1.29 | 0.283 | 0.75 | 0.43, 1.32 | 0.315 |
| 30-34 | 122 (16) | 174 (15) | 0.86 | 0.50, 1.49 | 0.595 | 0.87 | 0.50, 1.53 | 0.625 | 0.84 | 0.47, 1.54 | 0.576 | 0.86 | 0.47, 1.58 | 0.631 |
| 35+ | 46 (6.1) | 71 (6.0) | 0.80 | 0.42, 1.51 | 0.490 | 0.81 | 0.42, 1.55 | 0.529 | 0.83 | 0.42, 1.67 | 0.604 | 0.86 | 0.43, 1.75 | 0.686 |
| Missing | 99 (13) | 166 (14) |  |  |  |  |  |  |  |  |  |  |  |  |
| **Breastfeeding duration (mths)** |  |  |  |  |  |  |  |  |  |  |  |  |  |  |
| Never breastfed | 101 (13) | 179 (15) | — | — |  | — | — |  | — | — |  | — | — |  |
| <6 | 223 (30) | 319 (27) | 1.24 | 0.91, 1.69 | 0.180 | 1.22 | 0.89, 1.68 | 0.223 | 1.34 | 0.96, 1.90 | 0.091 | 1.32 | 0.93, 1.88 | 0.118 |
| 6-11 | 131 (17) | 200 (17) | 1.12 | 0.79, 1.58 | 0.541 | 1.11 | 0.78, 1.58 | 0.578 | 1.30 | 0.89, 1.91 | 0.180 | 1.30 | 0.88, 1.92 | 0.194 |
| 12-23 | 131 (17) | 244 (21) | 0.93 | 0.65, 1.32 | 0.675 | 0.86 | 0.60, 1.23 | 0.404 | 1.01 | 0.68, 1.48 | 0.973 | 0.93 | 0.63, 1.39 | 0.737 |
| 24+ | 72 (9.5) | 82 (6.9) | 1.49 | 0.95, 2.33 | 0.081 | 1.45 | 0.92, 2.29 | 0.108 | 1.57 | 0.96, 2.55 | 0.072 | 1.50 | 0.92, 2.47 | 0.106 |
| Missing | 97 (13) | 161 (14) |  |  |  |  |  |  |  |  |  |  |  |  |
| **Menopausal status** |  |  |  |  |  |  |  |  |  |  |  |  |  |  |
| Premenopausal | 242 (32) | 355 (30) | — | — |  | — | — |  | — | — |  | — | — |  |
| Postmenopausal | 513 (68) | 830 (70) | 0.75 | 0.51, 1.12 | 0.161 | 0.81 | 0.54, 1.20 | 0.296 | 0.82 | 0.53, 1.26 | 0.361 | 0.88 | 0.56, 1.36 | 0.557 |
| **Age at menopause** |  |  |  |  |  |  |  |  |  |  |  |  |  |  |
| <50 | 116 (15) | 185 (16) | — | — |  | — | — |  | — | — |  | — | — |  |
| 50-54 | 332 (44) | 570 (48) | 0.87 | 0.66, 1.16 | 0.345 | 0.90 | 0.68, 1.21 | 0.491 | 0.88 | 0.65, 1.20 | 0.416 | 0.91 | 0.66, 1.25 | 0.546 |
| 55+ | 64 (8.5) | 71 (6.0) | 1.52 | 0.98, 2.36 | 0.060 | 1.60 | 1.02, 2.50 | 0.040 | 1.51 | 0.93, 2.44 | 0.094 | 1.58 | 0.96, 2.58 | 0.070 |
| Missing | 243 (32) | 359 (30) |  |  |  |  |  |  |  |  |  |  |  |  |
| **MHT status** |  |  |  |  |  |  |  |  |  |  |  |  |  |  |
| Never | 192 (25) | 354 (30) | — | — |  | — | — |  | — | — |  | — | — |  |
| Former | 161 (21) | 281 (24) | 1.02 | 0.77, 1.35 | 0.914 | 1.02 | 0.76, 1.35 | 0.916 | 0.98 | 0.72, 1.33 | 0.878 | 0.97 | 0.71, 1.33 | 0.860 |
| Current | 157 (21) | 192 (16) | 1.50 | 1.12, 2.02 | 0.007 | 1.41 | 1.04, 1.91 | 0.025 | 1.55 | 1.12, 2.15 | 0.008 | 1.46 | 1.05, 2.03 | 0.025 |
| Missing | 245 (32) | 358 (30) |  |  |  |  |  |  |  |  |  |  |  |  |
| **Benign breast disease** |  |  |  |  |  |  |  |  |  |  |  |  |  |  |
| Never | 467 (62) | 831 (70) | — | — |  | — | — |  | — | — |  | — | — |  |
| Ever | 288 (38) | 354 (30) | 1.46 | 1.19, 1.78 | <0.001 | 1.36 | 1.11, 1.68 | 0.003 | 1.69 | 1.36, 2.11 | <0.001 | 1.59 | 1.26, 2.00 | <0.001 |
| **Family history of breast cancer** |  |  |  |  |  |  |  |  |  |  |  |  |  |  |
| No | 564 (75) | 931 (79) | — | — |  | — | — |  | — | — |  | — | — |  |
| Yes | 191 (25) | 254 (21) | 1.26 | 1.00, 1.57 | 0.047 | 1.26 | 1.00, 1.58 | 0.051 | 1.37 | 1.07, 1.75 | 0.013 | 1.35 | 1.05, 1.74 | 0.017 |
| **BMI at recruitment (kg/m²)** |  |  |  |  |  |  |  |  |  |  |  |  |  |  |
| <18.5 | 8 (1.1) | 3 (0.3) | 3.37 | 0.94, 15.8 | 0.080 | 4.07 | 1.09, 19.8 | 0.050 | 3.50 | 0.85, 18.2 | 0.099 | 4.13 | 0.97, 22.1 | 0.068 |
| 18.5-25 | 421 (56) | 550 (46) | — | — |  | — | — |  | — | — |  | — | — |  |
| 25-30 | 203 (27) | 405 (34) | 0.69 | 0.55, 0.86 | 0.001 | 0.74 | 0.58, 0.93 | 0.009 | 0.67 | 0.52, 0.85 | 0.001 | 0.71 | 0.55, 0.91 | 0.007 |
| 30+ | 116 (15) | 213 (18) | 0.78 | 0.59, 1.04 | 0.092 | 0.88 | 0.66, 1.19 | 0.416 | 0.73 | 0.53, 1.00 | 0.052 | 0.83 | 0.60, 1.15 | 0.272 |
| Missing | 7 (0.9) | 14 (1.2) |  |  |  |  |  |  |  |  |  |  |  |  |
| **BMI at age 20 (kg/m²)*** |  |  |  |  |  |  |  |  |  |  |  |  |  |  |
| <18.5 | 77 (10) | 72 (6.1) | 1.59 | 1.10, 2.28 | 0.012 | 1.65 | 1.14, 2.38 | 0.008 | 1.47 | 0.99, 2.19 | 0.059 | 1.50 | 1.00, 2.25 | 0.049 |
| 18.5-25 | 506 (67) | 828 (70) | — | — |  | — | — |  | — | — |  | — | — |  |
| 25+ | 39 (5.2) | 69 (5.8) | 1.04 | 0.66, 1.60 | 0.875 | 1.13 | 0.72, 1.77 | 0.580 | 1.06 | 0.65, 1.70 | 0.811 | 1.16 | 0.71, 1.87 | 0.559 |
| Missing | 133 (18) | 216 (18) |  |  |  |  |  |  |  |  |  |  |  |  |
| **Physical activity (MET h/wk)** * |  |  |  |  |  |  |  |  |  |  |  |  |  |  |
| <9 | 153 (20) | 271 (23) | — | — |  | — | — |  | — | — |  | — | — |  |
| 9-17 | 145 (19) | 246 (21) | 1.02 | 0.76, 1.37 | 0.903 | 0.97 | 0.72, 1.32 | 0.855 | 0.91 | 0.66, 1.26 | 0.569 | 0.87 | 0.62, 1.21 | 0.393 |
| 18+ | 451 (60) | 660 (56) | 1.18 | 0.93, 1.51 | 0.174 | 1.16 | 0.90, 1.48 | 0.251 | 1.21 | 0.93, 1.58 | 0.161 | 1.16 | 0.88, 1.52 | 0.284 |
| Missing | 6 (0.8) | 8 (0.7) |  |  |  |  |  |  |  |  |  |  |  |  |
| **Alcohol units per week** |  |  |  |  |  |  |  |  |  |  |  |  |  |  |
| 0 | 149 (20) | 258 (22) | — | — |  | — | — |  | — | — |  | — | — |  |
| 1-9 | 200 (26) | 317 (27) | 1.04 | 0.78, 1.38 | 0.785 | 1.04 | 0.78, 1.39 | 0.764 | 1.17 | 0.86, 1.60 | 0.314 | 1.18 | 0.86, 1.61 | 0.308 |
| 10-19 | 208 (28) | 290 (24) | 1.20 | 0.90, 1.60 | 0.205 | 1.21 | 0.91, 1.62 | 0.197 | 1.24 | 0.91, 1.70 | 0.171 | 1.22 | 0.89, 1.68 | 0.221 |
| 20-29 | 126 (17) | 190 (16) | 1.03 | 0.74, 1.42 | 0.865 | 1.04 | 0.75, 1.44 | 0.813 | 1.22 | 0.85, 1.74 | 0.277 | 1.22 | 0.85, 1.75 | 0.285 |
| 30+ | 72 (9.5) | 130 (11) | 0.88 | 0.60, 1.28 | 0.512 | 0.92 | 0.62, 1.34 | 0.651 | 0.89 | 0.59, 1.33 | 0.563 | 0.91 | 0.60, 1.38 | 0.670 |
| **Smoking status** |  |  |  |  |  |  |  |  |  |  |  |  |  |  |
| Never | 453 (60) | 709 (60) | — | — |  | — | — |  | — | — |  | — | — |  |
| Former | 261 (35) | 392 (33) | 1.05 | 0.85, 1.30 | 0.621 | 1.09 | 0.87, 1.35 | 0.458 | 1.04 | 0.83, 1.32 | 0.714 | 1.09 | 0.86, 1.38 | 0.489 |
| Current | 41 (5.4) | 82 (6.9) | 0.78 | 0.51, 1.17 | 0.228 | 0.78 | 0.51, 1.19 | 0.259 | 0.76 | 0.48, 1.19 | 0.234 | 0.78 | 0.49, 1.22 | 0.277 |
| Missing | 0 (0) | 2 (0.2) |  |  |  |  |  |  |  |  |  |  |  |  |
| **Mammographic density (quartiles)** |  |  |  |  |  |  |  |  |  |  |  |  |  |  |
| 1 (<11.45%) | 73 (9.7) | 225 (19) |  |  |  | — | — |  |  |  |  | — | — |  |
| 2 (11.45-22.00%) | 96 (13) | 202 (17) |  |  |  | 1.39 | 0.95, 2.03 | 0.094 |  |  |  | 1.42 | 0.94, 2.16 | 0.094 |
| 3 (22.00-26.70%) | 105 (14) | 193 (16) |  |  |  | 1.66 | 1.14, 2.44 | 0.009 |  |  |  | 1.58 | 1.04, 2.41 | 0.034 |
| 4 (>36.70%) | 127 (17) | 170 (14) |  |  |  | 2.13 | 1.44, 3.17 | <0.001 |  |  |  | 2.18 | 1.42, 3.38 | <0.001 |
| Missing | 354 (47) | 395 (33) |  |  |  |  |  |  |  |  |  |  |  |  |
| **Invasive status** |  |  |  |  |  |  |  |  |  |  |  |  |  |  |
| DCIS | 47 (6.2) | 261 (22) |  |  |  |  |  |  | — | — |  | — | — |  |
| Invasive | 708 (94) | 924 (78) |  |  |  |  |  |  | 1.50 | 0.85, 2.67 | 0.166 | 1.58 | 0.88, 2.84 | 0.128 |
| **Grade** |  |  |  |  |  |  |  |  |  |  |  |  |  |  |
| 1 | 88 (12) | 250 (21) |  |  |  |  |  |  | — | — |  | — | — |  |
| 2 | 335 (44) | 477 (40) |  |  |  |  |  |  | 1.64 | 1.20, 2.27 | 0.002 | 1.65 | 1.19, 2.28 | 0.003 |
| 3 | 264 (35) | 187 (16) |  |  |  |  |  |  | 2.86 | 1.97, 4.18 | <0.001 | 2.98 | 2.04, 4.39 | <0.001 |
| Missing | 68 (9.0) | 271 (23) |  |  |  |  |  |  |  |  |  |  |  |  |
| **Morphology type** |  |  |  |  |  |  |  |  |  |  |  |  |  |  |
| Ductal | 549 (73) | 698 (59) |  |  |  |  |  |  | — | — |  | — | — |  |
| Lobular | 95 (13) | 119 (10) |  |  |  |  |  |  | 0.92 | 0.65, 1.32 | 0.662 | 0.89 | 0.62, 1.27 | 0.529 |
| Mixed | 25 (3.3) | 43 (3.6) |  |  |  |  |  |  | 0.92 | 0.51, 1.61 | 0.771 | 0.88 | 0.49, 1.56 | 0.668 |
| Other | 39 (5.2) | 64 (5.4) |  |  |  |  |  |  | 0.95 | 0.57, 1.55 | 0.828 | 0.89 | 0.54, 1.47 | 0.656 |
| Missing | 47 (6.2) | 261 (22) |  |  |  |  |  |  |  |  |  |  |  |  |
| **Node status** |  |  |  |  |  |  |  |  |  |  |  |  |  |  |
| Negative | 304 (40) | 601 (51) |  |  |  |  |  |  | — | — |  | — | — |  |
| Positive | 234 (31) | 194 (16) |  |  |  |  |  |  | 1.89 | 1.44, 2.48 | <0.001 | 1.88 | 1.43, 2.49 | <0.001 |
| Missing | 217 (29) | 390 (33) |  |  |  |  |  |  |  |  |  |  |  |  |
| **Tumour size (mm)** |  |  |  |  |  |  |  |  |  |  |  |  |  |  |
| <21 | 336 (45) | 656 (55) |  |  |  |  |  |  | — | — |  | — | — |  |
| 21-50 | 225 (30) | 162 (14) |  |  |  |  |  |  | 2.01 | 1.52, 2.65 | <0.001 | 2.03 | 1.53, 2.70 | <0.001 |
| 50+ | 31 (4.1) | 15 (1.3) |  |  |  |  |  |  | 2.79 | 1.37, 5.85 | 0.005 | 2.64 | 1.29, 5.61 | 0.009 |
| Missing | 163 (22) | 352 (30) |  |  |  |  |  |  |  |  |  |  |  |  |
| **ER status** |  |  |  |  |  |  |  |  |  |  |  |  |  |  |
| Positive | 525 (70) | 791 (67) |  |  |  |  |  |  | — | — |  | — | — |  |
| Negative | 137 (18) | 87 (7.3) |  |  |  |  |  |  | 1.79 | 1.19, 2.70 | 0.005 | 1.78 | 1.17, 2.72 | 0.007 |
| Missing | 93 (12) | 307 (26) |  |  |  |  |  |  |  |  |  |  |  |  |
| **PR status** |  |  |  |  |  |  |  |  |  |  |  |  |  |  |
| Positive | 283 (37) | 375 (32) |  |  |  |  |  |  | — | — |  | — | — |  |
| Negative | 175 (23) | 154 (13) |  |  |  |  |  |  | 0.83 | 0.58, 1.19 | 0.313 | 0.80 | 0.55, 1.15 | 0.226 |
| Missing | 297 (39) | 656 (55) |  |  |  |  |  |  |  |  |  |  |  |  |
| **HER2 status** |  |  |  |  |  |  |  |  |  |  |  |  |  |  |
| Positive | 102 (14) | 92 (7.8) |  |  |  |  |  |  | — | — |  | — | — |  |
| Negative | 524 (69) | 725 (61) |  |  |  |  |  |  | 1.18 | 0.82, 1.70 | 0.381 | 1.11 | 0.76, 1.61 | 0.596 |
| Missing | 129 (17) | 368 (31) |  |  |  |  |  |  |  |  |  |  |  |  |
| ^1^n (%) | | | | | | | | | | | | | | |
| ^2^OR = Odds Ratio  ^3^CI = Confidence Interval | | | | | | | | | | | | | | |
| ^4^Model 1: Age at menarche, Menopause, Age at menopause, MHT status, Oral contraceptive use, Parity, Age at first birth, Breast feeding duration (mths), Benign breast disease, Family history of breast cancer, BMI at recruitment (kg/m²), BMI at age 20 (kg/m²), Physical activity (MET h/wk), Alcohol units per week, Smoking status, Age at Diagnosis, Time from Recruitment to BC diagnosis, Year of diagnosis | | | | | | | | | | | | | | |
| ^5^Model 2: Age at menarche, Menopause, Age at menopause, MHT status, Oral contraceptive use, Parity, Age at first birth, Breast feeding duration (mths), Benign breast disease, Family history of breast cancer, BMI at recruitment (kg/m²), BMI at age 20 (kg/m²), Physical activity (MET h/wk), Alcohol units per week, Smoking status, Age at diagnosis, Time from recruitment to BC diagnosis, Year of diagnosis, Percent mammographic density (quartiles), Time from mammographic density measurement to BRCA diagnosis | | | | | | | | | | | | | | |
| ^6^Model 3: Age at menarche, Menopause, Age at menopause, MHT status, Oral contraceptive use, Parity, Age at first birth, Breast feeding duration (mths), Benign breast disease, Family history of breast cancer, BMI at recruitment (kg/m²), BMI at age 20 (kg/m²), Physical activity (MET h/wk), Alcohol units per week, Smoking status, Age at diagnosis, Time from recruitment to BC diagnosis, Year of diagnosis, Invasive status, Morphology, Node status, Tumour size, ER status, PR status, HER2 status | | | | | | | | | | | | | | |
| ^7^Model 4: Age at menarche, Menopause, Age at menopause, MHT status, Oral contraceptive use, Parity, Age at first birth, Breast feeding duration (mths), Benign breast disease, Family history of breast cancer, BMI at recruitment (kg/m²), BMI at age 20 (kg/m²), Physical activity (MET h/wk), Alcohol units per week, Smoking status, Age at diagnosis, Time from recruitment to BC diagnosis, Year of diagnosis, Percent mammographic density (quartiles), Time from mammographic density measurement to BRCA diagnosis, Invasive status, Morphology, Node status, Tumour size, ER status, PR status, HER2 status | | | | | | | | | | | | | | |

**Supplementary Figure 1**: Hypothesised causal structure relating risk factors, breast density, tumour characteristics and mode of detection.

Directed acyclic graph (DAG) representing the assumed causal relationships underlying the case-case analysis. Nodes are positioned along the time axis at which each variable is measured: risk factors reflect lifelong exposures ascertained at recruitment; breast density is measured on the pre-diagnostic screening mammogram; tumour characteristics and mode of detection are ascertained at diagnosis. Breast density is temporally intermediate and may act as a mediator of risk factors that causally influence density (e.g. menopausal hormone therapy, body mass index) or as a confounder of risk factors merely correlated with density; tumour characteristics, being downstream of all risk factors, can act only as a mediator. Age/timing (age at diagnosis, time between recruitment/density measurement and diagnosis, calendar year of diagnosis) is a common cause and is adjusted for in all models. Because analyses are restricted to breast cancer cases, they are implicitly conditioned on disease occurrence (breast cancer occurrence node); this node is a collider, so restriction can induce non-causal associations between risk factors and density. The minimal sufficient adjustment set for the direct effect of a risk factor on mode of detection, derived from this DAG, is {age/timing, breast density, tumour characteristics}, corresponding to the fully-adjusted model (Model 4); adjustment for breast density additionally blocks the collider path opened by the case-only design.

**Supplementary Figure 2:** Distribution of mammographic percent density by mode of detection and tumour characteristics. Details in **Supplementary Table 2,** including test for differences adjusted and unadjusted by age at mammogram. Abbreviations: MHT = Menopausal Hormone Therapy, BMI = Body Mass Index, MET = Metabolic Equivalent of Task.

**Supplementary Figure 3:** Distribution of mammographic percent density by risk factors for breast cancer. Details and age adjusted analyses are shown in **Supplementary Table 3**, including test for differences adjusted and unadjusted by age at mammogram. Abbreviations: MHT = Menopausal Hormone Therapy, BMI = Body Mass Index, MET = Metabolic Equivalent of Task.

**Supplementary Figure 4**: Examples of timelines showing study entry when risk factors were assessed (R), mammographic density (MD) measurement, and breast cancer (BC) diagnosis, in relation to routine screening rounds every three years, for SDBC (**Panel A)** and IBC (**Panel B**). These examples show variation in timing of MD assessment relative to screening and diagnosis, including scenarios where MD is measured before, between, or after specific screening rounds in relation to BC diagnosis.

**Panel A:** Screen-detected breast cancers

**Panel B:** Interval breast cancers
